# High interleukin-6 levels induced by COVID-19 pneumonia correlate with increased circulating follicular helper T cell frequency and strong neutralization antibody response in the acute phase of Omicron breakthrough infection

**DOI:** 10.3389/fimmu.2024.1377014

**Published:** 2024-04-17

**Authors:** Hitoshi Kawasuji, Yoshitomo Morinaga, Kentaro Nagaoka, Hideki Tani, Yoshihiro Yoshida, Hiroshi Yamada, Yusuke Takegoshi, Makito Kaneda, Yushi Murai, Kou Kimoto, Hideki Niimi, Yoshihiro Yamamoto

**Affiliations:** ^1^ Department of Clinical Infectious Diseases, Toyama University Graduate School of Medicine and Pharmaceutical Sciences, Toyama, Japan; ^2^ Department of Microbiology, Toyama University Graduate School of Medicine and Pharmaceutical Sciences, Toyama, Japan; ^3^ Department of Virology, Toyama Institute of Health, Toyama, Japan; ^4^ Department of Clinical Laboratory and Molecular Pathology, Toyama University Graduate School of Medicine and Pharmaceutical Sciences, Toyama, Japan

**Keywords:** Omicron, pneumonia, neutralizing antibody, interleukin-6, circulating follicular helper T cell

## Abstract

**Background:**

Acute immune responses to coronavirus disease 2019 (COVID-19) are influenced by variants, vaccination, and clinical severity. Thus, the outcome of these responses may differ between vaccinated and unvaccinated patients and those with and without COVID-19-related pneumonia. In this study, these differences during infection with the Omicron variant were investigated.

**Methods:**

A total of 67 patients (including 47 vaccinated and 20 unvaccinated patients) who were hospitalized within 5 days after COVID-19 symptom onset were enrolled in this prospective observational study. Serum neutralizing activity was evaluated using a pseudotyped virus assay and serum cytokines and chemokines were measured. Circulating follicular helper T cell (cTfh) frequencies were evaluated using flow cytometry.

**Results:**

Twenty-five patients developed COVID-19 pneumonia on hospitalization. Although the neutralizing activities against wild-type and Delta variants were higher in the vaccinated group, those against the Omicron variant as well as the frequency of developing pneumonia were comparable between the vaccinated and unvaccinated groups. IL-6 and CXCL10 levels were higher in patients with pneumonia than in those without it, regardless of their vaccination status. Neutralizing activity against the Omicron variant were higher in vaccinated patients with pneumonia than in those without it. Moreover, a distinctive correlation between neutralizing activity against Omicron, IL-6 levels, and cTfh proportions was observed only in vaccinated patients.

**Conclusions:**

The present study demonstrates the existence of a characteristic relationship between neutralizing activity against Omicron, IL-6 levels, and cTfh proportions in Omicron breakthrough infection.

## Introduction

The Omicron (B.1.1.529) variant of the severe acute respiratory syndrome coronavirus 2 (SARS-CoV-2) was first reported on November 2021 (1, 2). This variant demonstrates higher transmission rates than previous variants, resulting in its rapid dominance worldwide (3, 4). Nonetheless, this variant causes milder symptoms and is associated with lower hospitalization rates and mortality (4). The decreased clinical severity of the Omicron variant may be due to the low fusogenicity of its spike (S) protein, leading to less tissue damage and restricted tropism of the virus in the upper respiratory tract of patients (due to altered transmembrane protease serine 2 [TMPRSS2] activity) (4–6).

The coronavirus disease 2019 (COVID-19) typically presents on chest computed tomography (CT) scans as peripheral bilateral ground-glass opacities (GGOs) or as multifocal rounded GGOs with or without consolidation and the reverse halo sign (7). However, compared with the Delta or precedent SARS-CoV-2 variants, the Omicron variant is characterized by the presence of fewer and less severe changes in chest CT data (8). Notably, vaccination attenuates the severity of COVID-19 pneumonia, as evident by the decreased incidence of typical CT findings (7). Nevertheless, the vaccine-induced protective effect against COVID-19 pneumonia lessens with time (9); thus, even vaccinated individuals develop pneumonia, and some also develop other severe and/or fatal manifestations (10). To date, few studies have examined the incidence of pneumonia during the Omicron wave, as well as the differences in immune responses between patients with *vs*. without pneumonia and between vaccinated *vs*. unvaccinated patients infected with Omicron variants (8, 10).

The neutralizing antibodies induced by vaccination or infection are important for protection from severe COVID-19 progression (11). Follicular helper T (Tfh) cells play an important role in inducing, producing, and maintaining high-affinity neutralizing antibodies against SARS-CoV-2 (12). The Tfh-mediated humoral immune responses differ between moderate and critically ill patients. A previous study reported that unvaccinated critically ill patients have lymph nodes and/or spleens with deficiently structured germinal centers and decreased numbers of Tfh cells (13), leading to delayed production of high-affinity neutralizing antibodies. Additionally, evaluation of unvaccinated patients infected with the ancestral Wuhan strain during the first wave of the COVID-19 pandemic revealed that the immune neutralization activity at hospitalization was significantly higher in patients with moderate disease than in those with severe-to-critical disease (14). These findings suggest that immune neutralization activities during the acute phase of infection are inversely correlated with disease severity and that delayed production of neutralizing antibodies is associated with severe COVID-19 progression. These results are consistent with other studies conducted during the same period (15).

Acute immune responses to COVID-19 are influenced by variants, vaccination, and clinical severity. The first aim of the present study was to assess the incidence of COVID-19 pneumonia and the differences in the neutralization activity or other immune characteristics during the acute phase of Omicron infection in vaccinated *vs*. unvaccinated patients. The second aim was to evaluate the differences in these acute immune responses in patients with *vs*. patients without pneumonia in both vaccinated and unvaccinated patients. As it can be difficult to assess the neutralization activity just before infection in vaccinated patients, we assessed the neutralization activity and serum levels of the markers of the protective status or immune response during the acute phase, namely, type I and III interferons (IFNs), interleukin (IL)-6, C-X-C motif ligand (CXCL)-10, and vascular endothelial growth factor (VEGF), within 5 days after symptom onset in both vaccinated and unvaccinated patients admitted to the hospital.

## Materials and methods

### Study design

This prospective, observational cohort study enrolled consecutive patients who were admitted to Toyama University Hospital with confirmed COVID-19 between January 2022 and March 2022. In the present study, the inclusion criteria were: age ≥18 years, newly diagnosed with COVID-19 based on positive reverse transcription polymerase chain reaction (RT-PCR) results, and admitted to the hospital within 5 days after symptom onset. The genomic survey of the local institute of public health during the study period reported that Omicron BA.1 variant was dominant, with a small population of Delta variant still being detected (16). After initial blood test and CT examination at admission, all patients received standard therapy depending on their clinical course and were followed until discharge or disease remission. Patients with clinical or laboratory evidence of prior COVID-19 infection and those who received the booster dose of vaccination prior to inclusion were excluded from the study.

### Study participants and protocol

Data on patient demographics, comorbidities, COVID-19 vaccine history, clinical presentation, laboratory findings, treatment regimens, and prognostic outcomes were collected from the medical charts. The participants were divided into two groups: those who had previously received two doses of BNT162b2 or mRNA-1273 vaccine (vaccinated group) and completely unvaccinated individuals (unvaccinated group). Breakthrough SARS-CoV-2 infection in the vaccinated group was defined as infection that occurred ≥14 days after the second dose of the monovalent BNT162b2 or mRNA-1273 vaccines (17).

Participants diagnosed with COVID-19 were asked to provide blood samples [serum, plasma, and peripheral blood mononuclear cells (PBMCs)] and nasopharyngeal swabs for viral load measurement at hospital admission. Serum, plasma, and PBMCs simultaneously collected were isolated and stored at −80°C until further analysis. Chest CT was also performed in all patients at admission. When a newly developed inflammatory lesion was detected, COVID-19 pneumonia was subsequently confirmed by trained pulmonary radiologists (KN and YY). The CT pattern of COVID-19 pneumonia was classified according to the dominant GGO pattern and pulmonary consolidations, as well as their extent and distribution, as follows: “extended/segmental GGO,” “extended/segmental organizing pneumonia-like,” and “others.” The diagnostic and COVID-19 pneumonia classification approaches were consistent with those described in previous reports (18). Hypoxemia requiring oxygen therapy was defined as a blood oxygen saturation (SpO_2_) level of ≤ 93% at rest/motion in room air, as defined previously (19). Patients without inflammatory lesions were confirmed to be negative for COVID-19 pneumonia.

### RT-qPCR analysis

Nasopharyngeal swabs and serum samples were collected, and RNA was extracted. The nasopharyngeal swab specimens were pretreated with 500 µL of sputazyme (Kyokuto Pharmaceutical, Tokyo, Japan). After centrifugation at 20,000×*g* for 30 min at 4°C, the supernatant was used for RNA extraction. A total of 60 µL RNA solution was obtained from 140 µL of the supernatant or 140 µL of serum using the QIAamp ViralRNA Mini Kit (Qiagen, Hilden, Germany) or Nippongene Isospin RNA Virus (Nippon Gene, Toyama, Japan), according to the manufacturers’ instructions. SARS-CoV-2 viral loads were quantified using *N2*-specific primer/probe sets and RT-qPCR analysis, according to the protocol of the National Institute of Infectious Diseases of Japan. Quantification quality was controlled using AcroMetrix COVID-19 RNA Control (Thermo Fisher Scientific, Waltham, MA, USA). The detection limit was of approximately 0.4 copies/µL (two copies/5 µL). RNAemia was defined as the presence of viral RNA in the serum, above the detection limit of the RT-qPCR assays (20).

Multiplex real-time one-step RT-PCR assays were also performed to detect mutations in the spike protein (L452R and G339D) using nucleic acids extracted from nasopharyngeal swabs. Testing was performed using a Light Cycler 96 Real-Time PCR System (Roche, Basel, Switzerland) along with Primer/Probe L452R Ver.2 and Primer/Probe G339D (Takara Bio, Shiga, Japan). All procedures were performed according to the manufacturers’ instructions. When the G339D mutation was detected and L452R was not, the Omicron BA.1 variant was identified in agreement with the local epidemic situation analyzed at the Toyama Institute of Health (16, 21).

### Blood samples

The stored blood serum, plasma, and PBMC samples of the enrolled patients were used for neutralization and serological assays, cytokine and RNAemia measurements, and phenotypic characterization of lymphocytes, as described in the following section. In our previous study (22), we performed preliminary experiments to assess the levels of inflammatory biomarkers at different time points in unvaccinated patients who developed pneumonia. We found that the levels of IFN-α, IL-6, and CXCL10 were decreased 5 days after the initial assessment. In another study we assessed neutralizing activity dynamics at different time points in unvaccinated patients (14), and found that the neutralization activities increased after 5 days and plateaued at 9–16 days after onset. Based on these earlier results, we here focused on immunoinflammatory biomarker levels and neutralizing activities within 5 days of symptom onset, which corresponds to the acute phase of SARS-CoV-2 infection (22, 23). Therefore, only blood samples collected within five days after symptom onset were used for this analysis.

### PBMC isolation and phenotype analysis

Whole blood samples (7 mL) were diluted with an equal volume of 0.9% NaCl solution and the PBMCs were isolated using Lymphoprep (Cosmo Bio, Tokyo, Japan). Briefly, after centrifugation at 800×*g* for 30 min at room temperature without the brake applied, the PBMC interface was carefully collected and washed with 0.9% NaCl via centrifugation at 250×*g* for 10 min. PBMC pellets were resuspended in 0.9% NaCl and washed again via centrifugation at 250×*g* for 10 min at room temperature. The cells were then divided into four tubes and cryopreserved at −80°C in Cell Banker 1 (Takara Bio) until analysis. After thawing, the cells were independently counted using a counting chamber (C-Chip DHC-B02; NanoEn Tek Inc., Seoul, Korea) with Türk stain. After washing twice with phosphate-buffered saline (PBS), the cells were fixed with Cell Cover (Anacyte Laboratories, Hamburg, Germany) and incubated with human TruStain FcX block (Biolegend, San Diego, CA, USA) for 10 min at room temperature in the dark. Next, the cells were stained for phenotypic analysis using 5-fold dilution of specific fluorochrome conjugated antibodies (**Supplementary Table 1**). Data acquisition was performed using FACS Celesta system (BD Biosciences, San Jose, CA, USA). PBMCs were isolated from healthy individuals who had received three doses of COVID-19 vaccination 2 weeks prior and then used as an internal positive control. A fluorescence minus one (FMO) control was used for gating analyses to distinguish positively from negatively stained cell populations.

### Cytokine measurement

Serum cytokines and chemokines—IFN-α, IFN-λ1 (IL-29), IFN-λ3 (IL-28B), IL-6, CXCL10, and VEGF—were measured using commercially available enzyme-linked immunosorbent assay kits, according to the manufacturers’ instructions (**Supplementary Table 2**). Each sample was measured on the first thaw. If an analyte signal was below the background signal, it was set to 0; if the signal was detectable but below the manufacturer’s lower limit of quantification, it was set to the lower limit of detection.

### Pseudotyped virus neutralization assay

Vesicular stomatitis virus (VSV) pseudotype bearing SARS-CoV-2 S protein was generated as previously described (24). The expression plasmid for the truncated S protein of SARS-CoV-2 variants, pCAGG-pm3-SARS2-Shu-d19-B1.617.2 (Delta-derived variant), pCAGG-pm3-SARS2-Shu-d19-B1.1.529.1 (Omicron BA.1-derived variant), and VSVs bearing envelope (VSV-G) were provided by Drs. C. Ono and Y. Matsuura of the Research Institute for Microbial Diseases, Osaka University, Japan. The pseudotyped VSVs were stored at −80°C until subsequent use. The neutralizing effects of each sample against pseudotyped VSVs were examined using a high-throughput chemiluminescent reduction neutralizing test (htCRNT), as previously described (25, 26). Briefly, serum samples were diluted 100-fold with Dulbecco’s modified Eagle’s medium (Nacalai Tesque, Kyoto, Japan) containing 10% heat-inactivated fetal bovine serum and were incubated with pseudotyped SARS-CoV-2 for 1 h. Afterward, VeroE6/TMPRSS2 cells (JCRB1819) were treated with medium-containing serum and pseudotyped virus mixture. The infectivity of the pseudotyped viruses was determined by measuring the luciferase activity after 24 h of incubation at 37°C. Samples without pseudotyped virus and those with pseudotyped virus but not serum were defined as positive (0%) and negative (100%) infection neutralizing controls, respectively.

### Serologic assays

Two different electrochemiluminescent immunoassays—Elecsys anti-SARS-CoV-2 immunoassay using recombinant nucleocapsid (N) antigen and Elecsys anti-SARS-CoV-2 S immunoassay using S protein receptor binding domain (RBD) (Roche)—were performed using plasma samples collected at admission to determine anti-N and anti-RBD antibodies in all patients. According to the manufacturer, a result was considered positive if the cutoff index was ≥1.0 and ≥0.8 U/mL for anti-N and anti-RBD, respectively. The lower and upper limits of quantification were 0.4 and 25,000.0 U/mL, respectively. The presence of anti-N antibodies indicated post-infection immunity, but it could also reflect acute humoral immune response, as a previous study reported that seroconversion for anti-N occurs significantly faster than that for anti-S in COVID-19 patients (27). Therefore, patients who were both anti-N and anti-RBD antibody positive were regarded as prior infected patients and excluded from the analysis.

### Statistical analysis

The participant’s medical and demographic characteristics were summarized as medians (interquartile ranges) or numbers (percentages). Differences between the two groups were tested using the Mann–Whitney U or Fisher’s exact tests. Correlations between the test findings were expressed using Pearson’s correlation coefficient. Analysis of the association between neutralizing activities and immune parameters are summarized in a correlation matrix. Statistical significance was defined as *P*<0.05. Statistical analysis and figure construction were performed using JMP Pro version 17.0.0 software (SAS Institute Inc., Cary, NC, USA) and GraphPad Prism version 9.5.1 (GraphPad Software, San Diego, CA, USA).

## Results

### Characterization of the study cohort

A total of 67 patients were included in the study, among whom 47 patients were vaccinated. All patients in the vaccinated group were infected 3–9 months after receiving the second dose of the BNT162b2 or mRNA-1273 vaccines. The demographic and clinical characteristics of the study cohort are summarized in **Table 1**. Patient age was higher in the vaccinated group than in the unvaccinated group, but the difference did not reach statistical significance. Sex, underlying diseases, body mass index, and febrile period were not significantly different between the vaccinated and unvaccinated groups. Sera and plasma were collected from all patients, but nasopharyngeal swabs were not collected in 4 vaccinated and 5 unvaccinated patients, and PBMCs were not collected in 11 vaccinated and 3 unvaccinated patients.

**Table 1:** Clinical characteristics of the patients with COVID-19.

Characteristics	Total (*n*=67)	Vaccinated group (*n*=47)	Unvaccinated group (*n*=20)	*P*-value
Demographics				
Age (years)	62 (49–73)	63 (51–74)	56 (44–69)	0.056
Sex (males)	46 (68.7%)	33 (70.2%)	13 (65.0%))	0.78
Underlying disease				
None	31 (46.3%)	24 (51.1%)	7 (35.0%)	0.29
Hypertension	29 (43.3%)	21 (44.7%)	8 (40.0%)	0.79
Diabetes mellitus	11 (16.4%)	8 (17.0%)	3 (15.0%)	>0.99
Respiratory disease	10 (14.9%)	5 (10.6%)	5 (25.0%)	0.15
Body mass index (kg/m^2^)	25.0 (22.0–27.6)	25.6 (22.3–27.8)	24.5 (21.3–27.0)	0.21
Febrile period (days)	4 [3–7]	4 [3–6]	5.5 [3–7]	0.11
Initial nasopharyngeal-viral load (log_10_ copies/μL)	4.4 (3.9–5.0)	4.4 (4.1–5.0)	4.3 (3.7–5.3)	0.60
RNAemia	10 (14.9%)	6 (12.8%)	4 (20.0%)	0.47
Treatment				
Untreated	35 (52.2%)	27 (57.4%)	8 (40.0%)	0.29
Remdesivir+Dexamethasone	6 (9.0%)	5 (10.6%)	1 (5.0%)	0.66
Anti-SARS-CoV-2 monoclonal antibodies	25 (37.3%)	14 (29.8%)	11 (55.0%)	0.060
Outcome				
30 days-mortality	0 (0%)	0 (0%)	0 (0%)	>0.99

Data are presented as n (%) or median (interquartile range).

The initial nasopharyngeal viral load at admission and the proportion of RNAemia were not significantly different between the vaccinated and unvaccinated groups (**Table 1**). Notably, RT-PCR assays revealed that all patients were infected with Omicron BA.1 variant, except for two patients in whom G339D was undetectable due to low viral loads (5.08 copies/μL and undetectable). Anti-N antibodies were undetectable in vaccinated patients, whereas unvaccinated patients were negative for anti-RBD antibodies at admission. Only two unvaccinated patients with pneumonia had low anti-N antibodies (3.66 and 4.16 cutoff index), but without anti-RBD antibodies, suggesting that these were likely early reactions to their current COVID-19 infection (27).

The incidence and characteristics of COVID-19 pneumonia in the vaccinated and unvaccinated groups are summarized in **Table 2**. Overall, 16 (34.0%) of the 47 vaccinated and 9 (45.0%) of the 22 unvaccinated patients presented with pneumonia at admission. The dominant CT pattern was segmental GGOs followed by extended GGOs, both in the vaccinated and unvaccinated groups. Five vaccinated patients and 1 unvaccinated patient developed hypoxemic respiratory failure at admission, whereas 2 vaccinated and 1 unvaccinated patient developed this complication within 5 days after admission. Only two vaccinated patients required invasive positive pressure ventilation, but all patients included in this study survived COVID-19, at least until 30 days after symptoms onset. All treatments, including antiviral medication with remdesivir, corticosteroids, and anti-SARS-CoV-2 monoclonal antibodies, were administered after the collection of the blood and nasopharyngeal swabs samples. Importantly, patient age, sex, underlying diseases, and body mass index were not significantly different between patients with and without pneumonia or hypoxemic respiratory failure, regardless of their vaccination status.

**Table 2:** Radiological characteristics of the patients with COVID-19.

Characteristics	Total (*n*=67)	Vaccinated group (*n*=47)	Unvaccinated group (*n*=20)	*P*-value
Pneumonia				
Present of pulmonary lesion	25 (37.3%)	16 (34.0%)	9 (45.0%)	0.42
Dominant CT pattern				
Extended GGO	6 (24.0%)	3 (18.8%)	3 (33.3%)	0.63
Segmental GGO	14 (56.0%)	9 (56.3%)	5 (55.6%)	>0.99
Extended OP-like	2 (8.0%)	2 (12.5%)	0 (0.0%)	0.52
Segmental OP-like	1 (1.8%)	0 (0.0%)	1 (11.1%)	0.36
Others	2 (8.0%)	2 (12.5%)	0 (0.0%)	0.52
Respiratory failure	9 (13.4%)	7 (14.9%)	2 (10.0%)	0.71
Onset				
At admission	6 (9.0%)	5 (10.6%)	1 (5.0%)	0.66
Within 5 days after admission	3 (4.5%)	2 (4.3%)	1 (5.0%)	>0.99
Oxygen therapy				
Required oxygen therapy	9 (13.4%)	7 (14.9%)	2 (10.0%)	0.71
IPPV	2 (3.0%)	2 (4.3%)	0 (0.0%)	>0.99
ECMO	1 (1.5%)	1 (2.1%)	0 (0.0%)	>0.99
Outcome				
30 days-mortality	0 (0.0%)	0 (0.0%)	0 (0.0%)	>0.99

Data are presented as n (%).

CT, computed tomography; GGO, ground-glass opacity; OP, organizing pneumonia; IPPV, intermittent positive pressure ventilation; ECMO, extracorporeal membrane oxygenation.

### Neutralization activity, anti-RBD antibody, and immunoinflammatory biomarker levels

**Figure 1:**
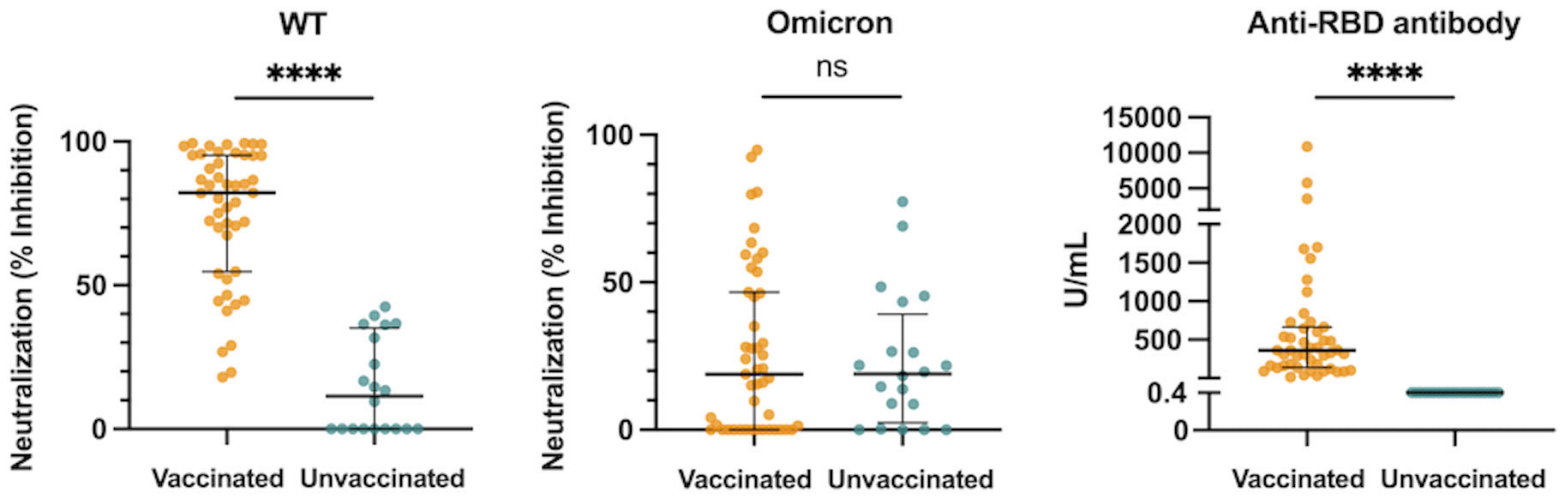
Neutralization activity against the WT- and Omicron-derived variants, and anti-RBD antibody levels at hospital admission (within 5 days after symptom onset) in vaccinated (n = 47) and unvaccinated patients (n = 20). The Mann-Whitney test was used to compare values between vaccinated and unvaccinated patients. Each dot represents an individual value. Bars indicate medians with interquartile ranges. WT, wild-type; RBD, receptor-binding domain; ****P<0.0001; ns, not significant.

The median htCRNT values of the wild-type- and Delta-derived variants, and the median anti-RBD antibody levels in the vaccinated group were 82.2% (interquartile range: 54.7–95.2%), 33.0% (23.0–65.9%), and 360.0 U/mL (139.0–662.0 U/mL), respectively, whereas in the unvaccinated group the values were all negative (<50% inhibition and <0.4 U/mL, respectively) (**Figure 1**; **Supplementary Figure 1A**). Although these htCRNT values were all significantly higher in vaccinated patients than in unvaccinated patients, the htCRNT values for Omicron BA.1 were similar (18.9% [0.0–46.6%] *vs.* 19.0% [2.4–39.2%]) (**Figure 1**). Only 2 unvaccinated patients with pneumonia had positive neutralizing antibodies against the Omicron variant (htCRNT values ≥50%), but without anti-RBD antibodies, suggesting that these were likely early reactions to their current COVID-19 infection. Moreover, the htCRNT values of the wild-type-, Delta-, and Omicron-derived variants were shown to be correlated with the anti-RBD antibody levels in the vaccinated group (**Supplementary Figure 1B**).

All measured proinflammatory cytokines and chemokines (IFN-α, IFN-λ1, IFN-λ3, IL-6, and CXCL10) levels and laboratory findings [neutrophil-to-lymphocyte ratio (NLR), lactate dehydrogenase (LD), and C-reactive protein (CRP)] in the acute phase were not significantly different between the vaccinated and unvaccinated groups (**Supplementary Figure 2**). Only the VEGF levels were significantly higher in the vaccinated patients than the unvaccinated patients (**Supplementary Figure 2**).

The association between neutralization activity or anti-RBD antibody levels and the initial nasopharyngeal viral loads at admission were also investigated. The htCRNT values of the wild-type- and Delta-derived variants were significantly correlated with the nasopharyngeal viral loads in the vaccinated patients, but not in unvaccinated patients (**Supplementary Figure 3**). The htCRNT values of the Omicron-derived variant and the anti-RBD antibody levels were not significantly correlated with the nasopharyngeal viral loads in either the vaccinated or unvaccinated patients. The correlation between the initial nasopharyngeal viral load and anti-RBD antibody levels in the unvaccinated patients is not shown in **Supplementary Figure 3** because the anti-RBD antibody levels in the unvaccinated patients were all negative (<0.4 U/mL).

### Association between acute immune response and the incidence of pneumonia

**Figure 2:**
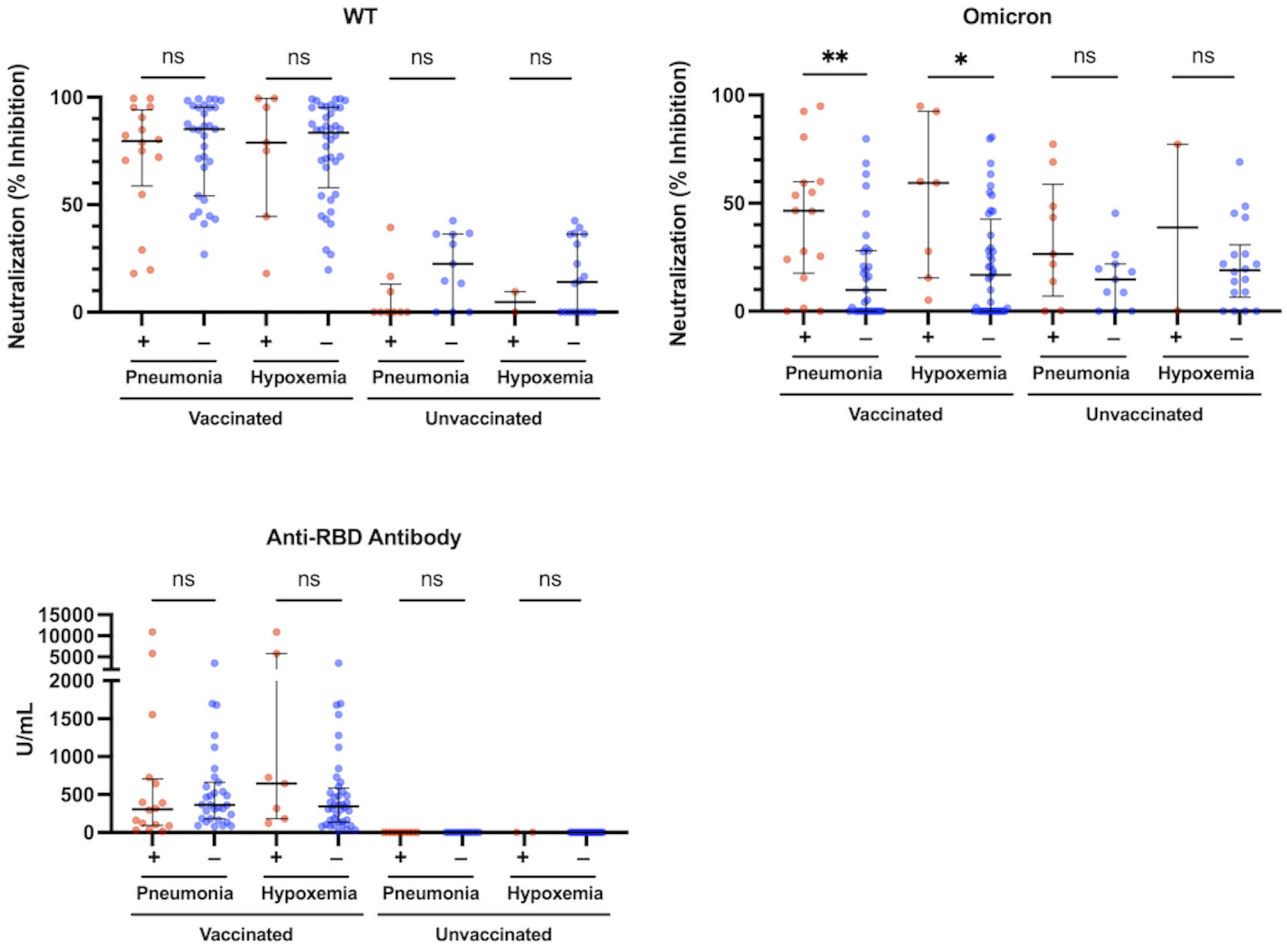
Neutralization activity against the WT- and Omicron-derived variants, and anti-RBD antibody levels at the acute phase of COVID-19, and associations with pneumonia or hypoxemic respiratory failure in vaccinated (n = 47) and unvaccinated patients (n = 20). The Mann-Whitney test was used to compare values between vaccinated patients with (n = 16) and without pneumonia (n = 31), vaccinated patients with (n = 7) and without hypoxemic respiratory failure (n = 40), unvaccinated patients with (n = 9) and without pneumonia (n = 11), and unvaccinated patients with (n = 2) and without hypoxemic respiratory failure (n = 18). Each level was measured at admission (within 5 days after symptom onset) and each dot represents an individual value. Bars indicate medians with interquartile ranges. WT, wild-type; RBD, receptor-binding domain; *P<0.05; **P<0.01; ns, not significant.

We analyzed the association between each of neutralization activity, antibody levels, and immunoinflammatory biomarkers and the presence of pneumonia in the vaccinated and unvaccinated groups. The htCRNT values for the wild-type- and Delta-derived variants and the anti-RBD antibody levels were not significantly different between patients with and without pneumonia in either the vaccinated or unvaccinated groups (**Figure 2**; **Supplementary Figure 4**). In contrast, the htCRNT values for the Omicron BA.1-derived variant were significantly higher in vaccinated patients with pneumonia than in those without pneumonia [46.5% (17.6–59.9%) vs. 9.8% (0.0–28.0%)] (**Figure 2**). This difference was also observed when comparing vaccinated patients with and without hypoxemic respiratory failure [59.4% (15.5–92.5%) vs. 16.9% (0.0–42.6%)]. On the other hand, the htCRNT values for the Omicron BA.1-derived variant were not significantly different between patients with and without pneumonia or between those with and without respiratory failure in the unvaccinated group (**Figure 2**).

**Figure 3:**
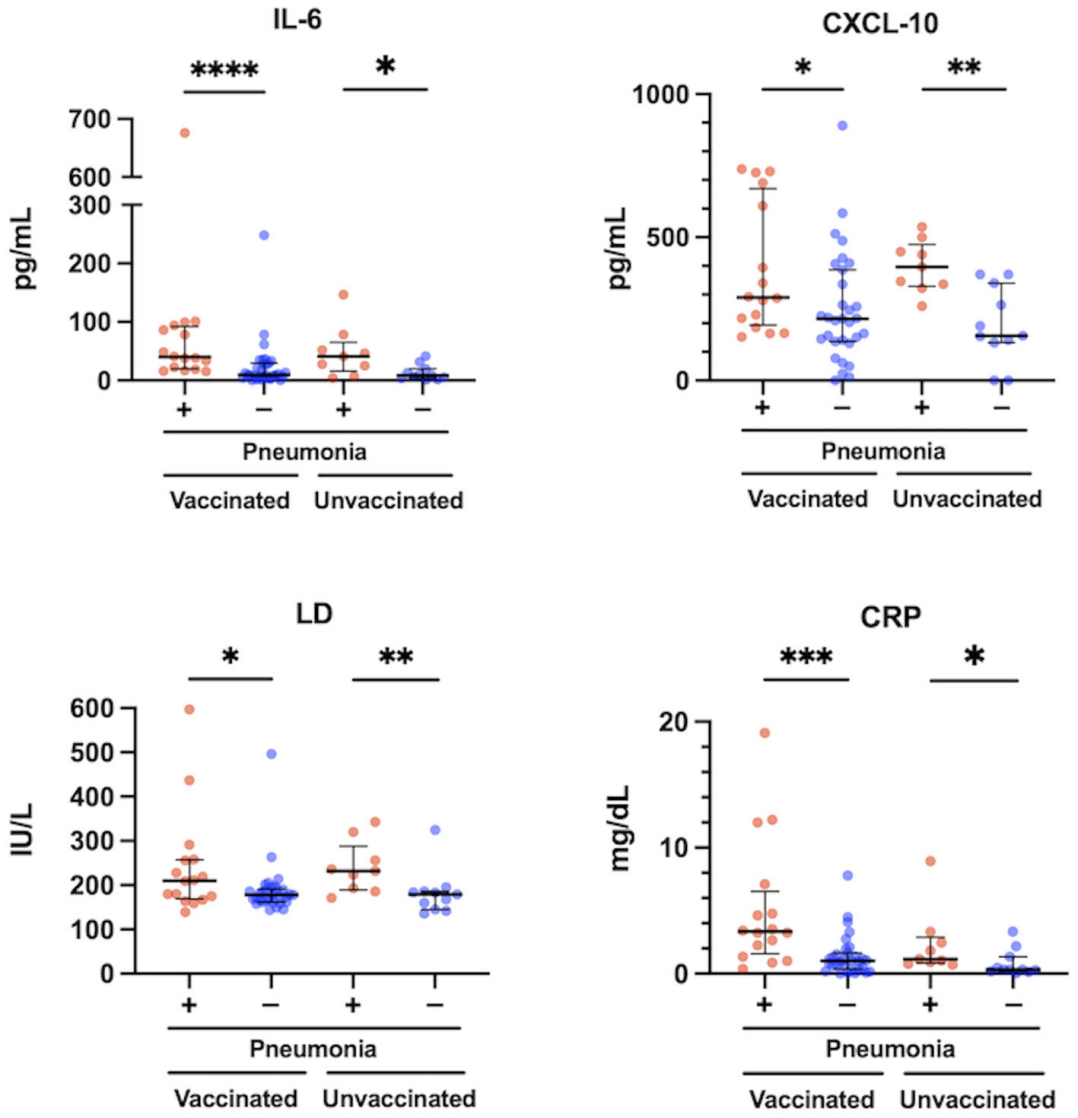
Relationship between serum levels of IL-6, CXCL10, LD and CRP, and pneumonia presence at the acute phase of COVID-19 in vaccinated (n = 47) and unvaccinated patients (n = 20). The Mann-Whitney test was used to compare values between vaccinated patients with (n = 16) and without pneumonia (n = 31), and unvaccinated patients with (n = 9) and without pneumonia (n = 11). Each level was measured at admission (within 5 days after symptom onset) and each dot represents an individual value. Bars indicate medians with interquartile ranges. IL, interleukin; CXCL10, C-X-C motif chemokine ligand 10; LD, lactate dehydrogenase; CRP, C-reactive protein; *P<0.05; **P<0.01; ***P<0.001; ****P<0.0001.

The levels of IL-6, CXCL10, LD, and CRP were significantly higher in patients with pneumonia than in those without pneumonia, both in the vaccinated and the unvaccinated group (**Figure 3**). The levels of IFN-α, IFN-λ1, IFN-λ3, VEGF, and NLR were not significantly different between these patients (**Supplementary Figure 4**). In the comparison between vaccinated and unvaccinated patients with pneumonia, the levels of IL-6, CXCL10, LD, IFN-α, IFN-λ1, IFN-λ3, VEGF, and NLR were not different (**Figure 3**; **Supplementary Figure 4**). In contrast, it was expected that the levels of CRP and the htCRNT values for the Delta-derived variant would be higher in the vaccinated patients with pneumonia than in the unvaccinated patients with pneumonia; however, these differences were also not statistically significant (*P*>0.05).

### Correlations among neutralization activity and immunoinflammatory biomarkers levels

**Figure 4:**
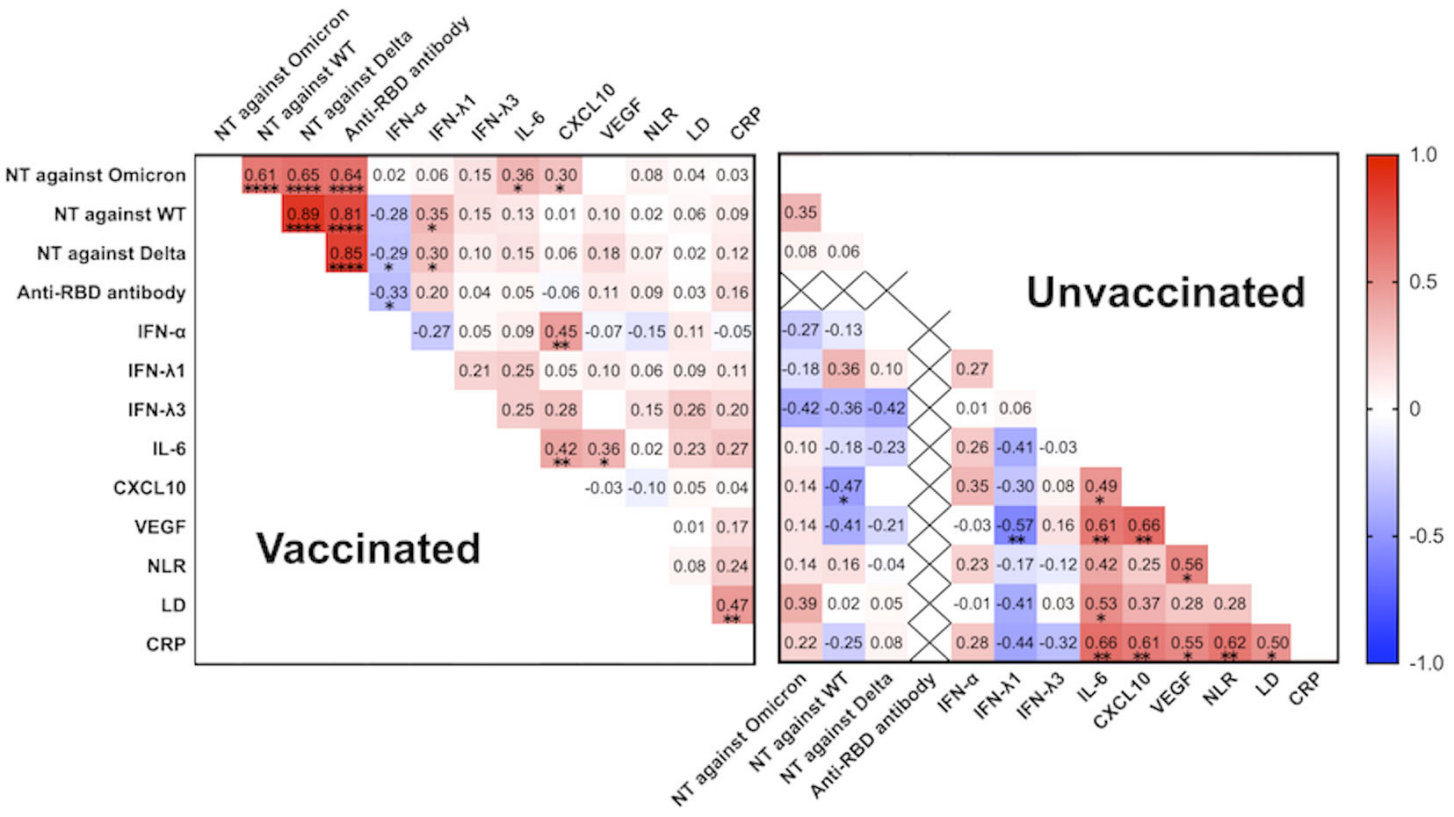
Correlation matrix of neutralizing activity against the wild-type-, Delta-, and Omicron-derived variants, anti-RBD antibody levels, and biomarker levels in vaccinated (n = 47) and unvaccinated patients (n = 20) at the acute phase of COVID-19 (within 5 days after symptom onset). Spearman correlation coefficients are plotted. Cells were colored according to the strength and trend of the correlations (shades of red = positive correlations, shades of blue = negative correlations). Anti-RBD antibody levels in the unvaccinated group were all negative (<0.4 U/mL). NT, neutralizing activity; WT, wild-type; RBD, receptor-binding domain; IFN, interferon; IL, interleukin; CXCL10, C-X-C motif chemokine ligand 10; VEGF, vascular endothelial growth factor; NLR, neutrophil-to-lymphocyte ratio; LD, lactate dehydrogenase; CRP, C-reactive protein; *P<0.05; **P<0.01; ****P<0.0001.

Among the vaccinated group, not only the htCRNT values of the wild-type- and Delta-derived variants, and anti-RBD antibody, but also those of IL-6 (*r*=0.36; *P*=0.01) and CXCL10 (*r*=0.30; *P*=0.042) levels were significantly correlated with the htCRNT values of the Omicron-derived variant (**Figure 4**). Among unvaccinated patients, the IL-6 levels were not correlated with the htCRNT values of the Omicron-derived variant. There were significant correlations between IL-6 levels and those of CXCL10 and VEGF in vaccinated patients, and between the IL-6, CXCL10, VEGF, LD, and CRP levels in the unvaccinated group (**Figure 4**).

### Subpopulation analysis of Tfh cells

**Figure 5:**
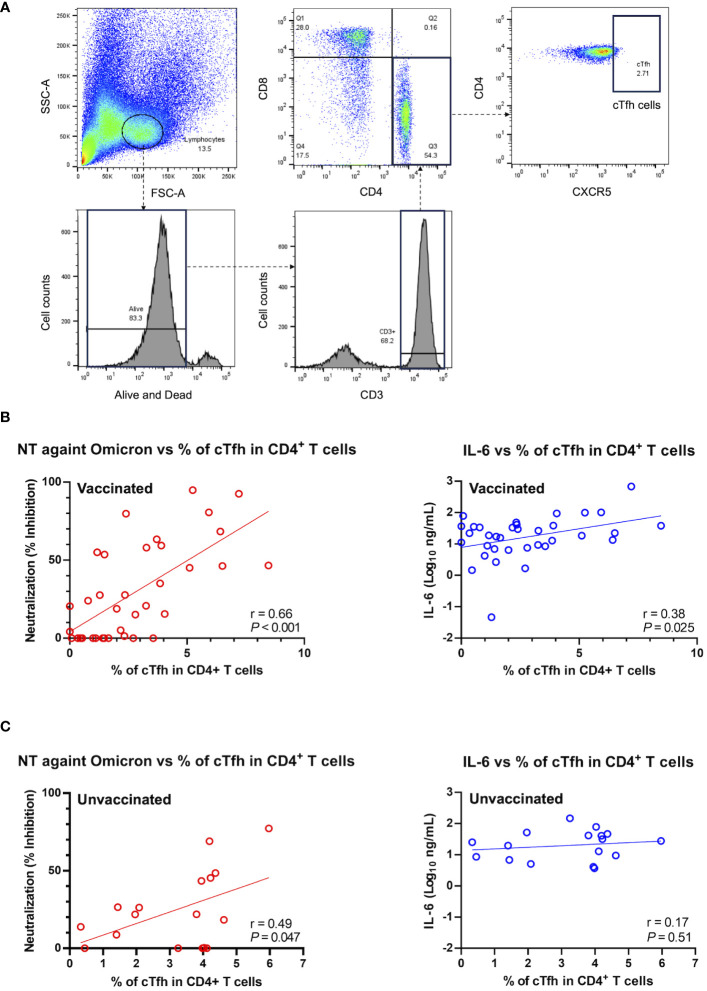
Relationship between neutralization activity against the Omicron-derived variant and IL-6, and the frequency of circulating follicular helper T cells in the CD4^+^ T cell population in vaccinated (n = 36) and unvaccinated patients (n = 17). **(A)** Representative flow cytometry plots of the gating strategy used to identify CXCR5^+^CD4^+^ cTfh cells. **(B, C)** Correlations between the cTfh cell population frequency and the neutralization activity against the Omicron-derived variant (red) or IL-6 levels (blue) in vaccinated (n = 37) **(B)** and unvaccinated patients (n = 17) **(C)**. The Pearson correlation was calculated, and the *P* value and r value are shown. The general linear regression lines are shown. Each level was measured at admission (within 5 days after symptom onset) and each dot represents an individual value. cTfh, circulating follicular helper T; NT, neutralizing activity; IL, interleukin.

To further investigate the potential factor contributing to the observed positive correlation between IL-6 and the htCRNT value of the Omicron-derived variant in the vaccinated group, the levels of CD3^+^CD4^+^CD8^−^CXCR5^+^ circulating Tfh (cTfh) cells were analyzed (**Figure 5A**). An internal positive control and FMO control were used for gating analyses to distinguish positively from negatively stained cell populations (**Supplementary Figure 5**). The frequency of CD3^+^CD4^+^CD8^−^CXCR5^+^ cTfh cells within the CD4^+^ cell population was linearly correlated with the htCRNT values of the Omicron-derived variant and IL-6 levels in vaccinated patients (**Figure 5B**). Noteworthily, the frequency of these CD3^+^CD4^+^CD8^−^CXCR5^+^ cTfh cells was only correlated with the Omicron-derived variant htCRNT values, but not with IL-6 levels, in the unvaccinated group (**Figure 5C**).

## Discussion

In the present study, which included patients vaccinated against SARS-CoV-2 3–9 months before infection, we found that the frequency and characteristics of COVID-19 pneumonia (18) were not significantly different between vaccinated and unvaccinated patients. Previous clinical studies have shown that vaccination significantly reduces the frequency and attenuates pneumonia severity, even during Omicron variant predominance (7, 28); however, older age and longer interval between the second vaccine shot and COVID-19 onset (≥6 months) were shown to increase pneumonia risk in breakthrough infections (28, 29). In the present study, the ages of the vaccinated patients were older (but not significantly) than those of the unvaccinated patients (**Table 1**), and all patients in the vaccinated group became infected 3–9 months after receiving the second dose of the BNT162b2 or mRNA-1273 vaccine. Thus, higher age in the vaccinated group and longer interval after the second vaccination were suggested to have potentially contributed to the similar frequencies of COVID-19 pneumonia between vaccinated and unvaccinated patients. Indeed, the neutralizing activity against Omicron variant, which was previously reported to correlate with the protection against pneumonia (30, 31), were not significantly different between vaccinated and unvaccinated patients. Similarly, the levels of all measured proinflammatory cytokines and chemokines (IFN-α, IFN-λ1, IFN-λ3, IL-6, and CXCL10) and the laboratory findings (NLR, LD, and CRP) in the acute phase were not significantly different between the vaccinated and unvaccinated groups, with the exception that the VEGF levels were significantly higher in vaccinated patients (**Supplementary Figure 2**). It is still unclear how we should interpret this difference; further investigation will be needed.

Acute immune responses to COVID-19 are influenced not only by vaccination, but also by variants and clinical severity. Contrary to the findings of previous studies that lower neutralization activity is associated with the severity and development of pneumonia in unvaccinated patients infected with the ancestral strain (14, 15), we found that the htCRNT values of the Omicron-derived variant during the acute infection phase were significantly higher in vaccinated patients with pneumonia or respiratory failure than in vaccinated patients without these conditions (**Figure 2**). Although the above difference was not observed among unvaccinated patients, positive neutralizing antibodies (htCRNT values ≥50%) without anti-RBD antibodies were observed in 2 unvaccinated patients with pneumonia, even within 5 days after symptom onset, suggesting that these were likely early reactions to their current Omicron infection (27) and that acute immune responses induced by pneumonia may be faster in individuals infected with the Omicron variant than in those infected with the ancestral strain (14, 15).

Our previous studies showed that serum IFN-α levels were higher in patients who developed pneumonia and hypoxemic respiratory failure, and were strongly predictive of hypoxemic respiratory failure in the early phase of COVID-19 due to Delta or precedent variants before Omicron emerged (22, 23). However, the present study demonstrated that serum levels of IFN-α were not significantly different between patients with and without pneumonia in either the vaccinated or unvaccinated patients (**Supplementary Figure 4**). On the other hand, serum levels of IL-6 were significantly higher in the vaccinated and unvaccinated patients with pneumonia than in those without pneumonia, respectively (**Figure 3**). Furthermore, a specific correlation between IL-6 levels and the htCRNT values for the Omicron-derived variant was observed in the vaccinated group, but not in the unvaccinated group (**Figure 4**).

IL-6 is a major contributor to the dysregulation of the immune response and plays an important role in cytokine release syndrome (commonly known as cytokine storm) related with severe COVID-19 (32). Indeed, elevated IL-6 levels were reported to be associated with systemic inflammation, pneumonia, hypoxemia, and poor prognosis (33), which agrees with our present findings that the levels of IL-6 were significantly higher in vaccinated or unvaccinated patients with pneumonia than in those without (**Figure 3**). Therefore, IL-6 inhibitors have been widely used as an effective treatment option in severe COVID-19 cases (34). Regarding the relationship between IL-6 and neutralizing antibodies, recent data revealed a significant reduction in anti-SARS-CoV-2 neutralizing antibody activity in recovered critically-ill patients treated with IL-6 and IL-1 inhibitors (35, 36).

IL-6 also regulates the early differentiation of antiviral Tfh cells, as well as the development of potent neutralizing antibodies (37). Tfh cells have an important role in T-cell-dependent B-cell response, as they support immunoglobulin class switching, germinal center-based affinity maturation, development of memory B cells, and long-lived humoral immunity (38). cTfh cells are representative of germinal center Tfh cells, as they share the surface expression of CXCR5, and account for approximately less than 10% of the CD4^+^ T cell compartment in the peripheral blood (12, 39). A previous study reported a correlation between SARS-CoV-2-specific cTfh cells and neutralizing antibody titers in COVID-19 patients (40). Although SARS-CoV-2-specific cTfh cells were not evaluated, the findings of the present study similarly demonstrated that the proportion of non-specific cTfh cells was correlated with the neutralizing activity against Omicron variant, both in the vaccinated and unvaccinated patients.

It remains unclear how a subset of activated CD4^+^ T cells can express the Tfh cell-defining CXCR5 marker, enter the B-cell follicles, mature further into germinal center Tfh cells, and promote the generation of high-affinity antibodies during the acute phase of the immune response. Previous studies reported that SARS-CoV-2 antibody titers are high in patients with pneumonia or oxygen requirement (39, 40), which agrees, to some degree, with our present findings that the neutralizing activities against the Omicron-derived variant were significantly higher in vaccinated patients with pneumonia than in those without pneumonia (**Figure 2**). Nonetheless, these studies only evaluated unvaccinated patients and antibody titers were consistently low before 7 days after onset (41). Indeed, in the present study evaluating the acute phase of the immune response, the neutralizing activities against the Omicron-derived variant were not significantly different between unvaccinated patients with and without pneumonia (**Figure 2**). More than 14 days after symptom onset, higher titers of SARS-CoV-2 antibodies were reported as a result of severe clinical manifestations of COVID-19 (42).

Recent studies showed that patients with Omicron variant breakthrough infections have robust recall humoral responses and pre-existing cellular immunity induced by vaccines, with activated cTfh cells rapidly increasing (12, 43). Based on these findings and on the specific correlations herein described between cTfh cell frequency, IL-6 levels, and neutralizing activity observed in vaccinated patients (**Figures 4**, **5**), it is reasonable to speculate that high IL-6 levels induced by COVID-19 pneumonia can rapidly promote the differentiation and recruitment of cTfh cells and, consequently, the production of high-affinity neutralizing antibodies against the Omicron variant in the early infection stages. Indeed, although the htCRNT values against the wild-type- and Delta-derived variants, and the anti-RBD antibody levels were not significantly different between vaccinated patients with and those without pneumonia, the neutralizing activity against the Omicron variant was significantly higher in vaccinated patients with pneumonia than in those without pneumonia, even within 5 days after symptom onset (**Figure 2**; **Supplementary Figure 4**).

This study had several limitations. First, as the study was performed at a single hospital and comprised a relatively small sample size, selection bias may have occurred. In addition, the number of unvaccinated patients was limited since the national vaccination campaign was widely applied, with most of the population being already vaccinated during the study period (when the Omicron variant was dominant). Second, we did not stain B cells/plasma cells makers, inducible costimulatory (ICOS), and programmed cell death protein 1 (PD-1), simultaneously. However, CXCR5 is reported to be the defining marker for cTfh cells (44), and ICOS and PD-1 staining seem not essential for distinguishing cTfh cells. Third, other cytokines such as IL-21 were not measured and their contribution to the promotion of cTfh differentiation and recruitment was not evaluated. Forth, the germinal center and SARS-CoV-2-specific Tfh cells, which were reported to be associated with the neutralizing activity (45), were not evaluated. Nonetheless, the cTfh cells were found to be correlated with the neutralizing activity in the present study; thus, cTfh cells may potentially be used as an alternative indicator of GC Tfh cells, but further studies are warranted. Considering our consistent results and the detected relationship between neutralizing activity against Omicron, IL-6 levels, and cTfh proportions, we believe that these limitations were not likely to have meaningfully affected our findings, which elucidated the differences in acute immune dynamics to Omicron infection between vaccinated and unvaccinated, with and without pneumonia, and between Omicron and the precedent variants before Omicron emerged.

## Conclusion

In conclusion, to the best of our knowledge, the present study is the first to demonstrate the characteristic relationship between neutralizing activity, IL-6 levels, and cTfh cell frequency in vaccinated (but not in unvaccinated) patients. The neutralizing activity was found to be significantly higher in patients with vs. without pneumonia within 5 days after symptoms onset, a feature that was specific to breakthrough infections. Furthermore, IL-6, but not IFN-α, was associated with the development of pneumonia and respiratory failure, which differed from the features of infections with precedent variants before Omicron emerged. As a better understanding of immune responses is important for enhanced clinical care, further studies are needed to confirm and extend the findings of this study.

## Data availability statement

The original contributions presented in the study are included in the article/**Supplementary Material**. Further inquiries can be directed to the corresponding author.

## Ethics statement

The studies involving humans were approved by The Ethical Review Board of the University of Toyama (approval No.: R2019167). The studies were conducted in accordance with the local legislation and institutional requirements. The participants provided their written informed consent to participate in this study.

## Author contributions

HK: Conceptualization, Data curation, Formal analysis, Investigation, Methodology, Resources, Validation, Visualization, Writing – original draft, Writing – review & editing. YoM: Conceptualization, Data curation, Formal analysis, Funding acquisition, Investigation, Methodology, Project administration, Supervision, Validation, Visualization, Writing – original draft, Writing – review & editing. KN: Conceptualization, Formal analysis, Investigation, Methodology, Resources, Supervision, Validation, Writing – review & editing. HT: Funding acquisition, Methodology, Resources, Supervision, Writing – review & editing. YYo: Investigation, Writing – review & editing. HY: Investigation, Writing – review & editing. YT: Resources, Writing – review & editing. MK: Resources, Writing – review & editing. YuM: Resources, Writing – review & editing. KK: Resources, Writing – review & editing. HN: Funding acquisition, Investigation, Supervision, Writing – review & editing. YYa: Funding acquisition, Project administration, Supervision, Writing – review & editing.
