# Supplementary material for: High interleukin-6 levels induced by COVID-19 pneumonia correlate with increased circulating follicular helper T cell frequency and strong neutralization antibody response in the acute phase of Omicron breakthrough infection
Source: Front Immunol. 2024 Apr 17;15:1377014. doi: 10.3389/fimmu.2024.1377014 (PMC11061453; doi:10.3389/fimmu.2024.1377014)
Supplement: Supplementary file 1 [file DataSheet_1.docx]

**Supplemental Figures and Tables:**

**
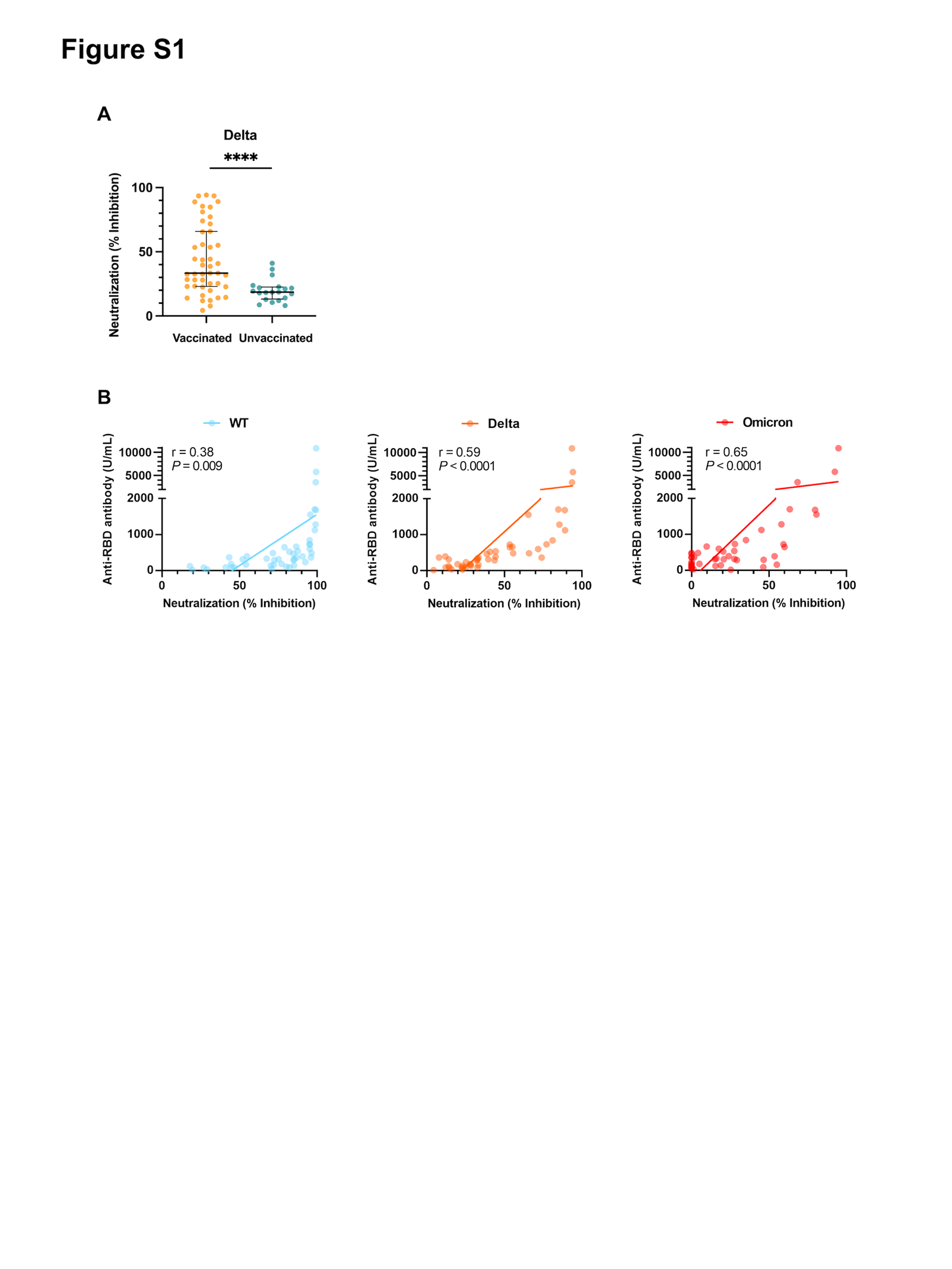
**

**Figure S1.** (**A**) Comparison of the neutralization activity against the Delta-derived variant in vaccinated (n = 47) and unvaccinated patients (n = 20). The Mann-Whitney test was used to compare values between the vaccinated and unvaccinated groups. (**B**) Correlation between the neutralization activity against the wild-type-, Delta-, and Omicron-derived variants, and anti-RBD antibody levels in the vaccinated group (n = 47). The Pearson correlation was calculated, and the *P* value and r value are shown. The general linear regression lines are shown.

Each level was measured at admission (within 5 days after symptom onset) and each dot represents an individual value (**A**, **B**). Bars indicate medians with interquartile ranges (**A**). RBD, receptor-binding domain; WT, wild-type; ****P<0.0001.

**
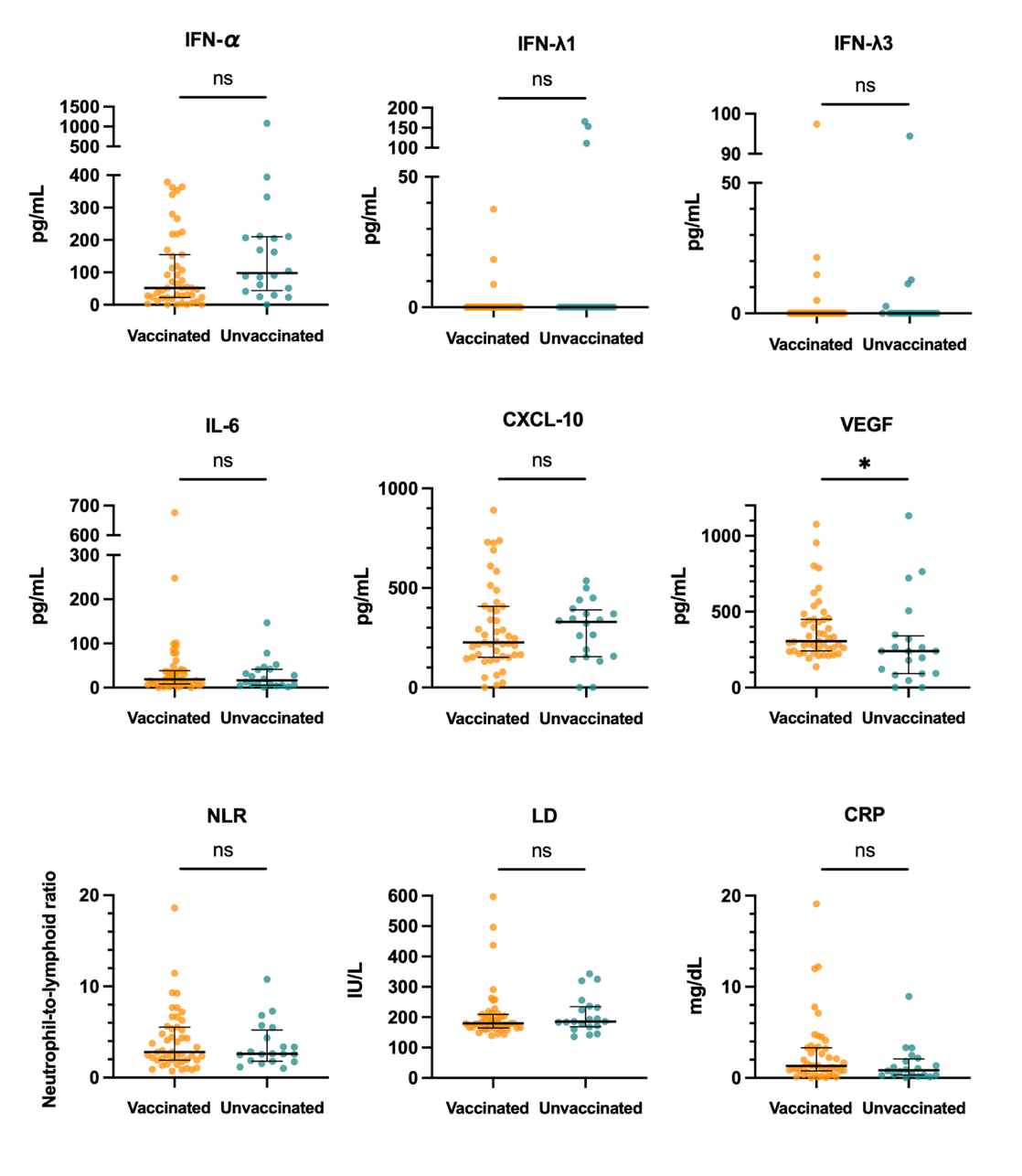
**

**Figure S2.** Serum biomarker levels at the acute phase of SARS-CoV-2 infection in vaccinated (n = 47) and unvaccinated patients (n = 20). The Mann-Whitney test was used to compare values between the vaccinated and unvaccinated groups. Each level was measured at admission (within 5 days after symptom onset) and each dot represents an individual value. Bars indicate medians with interquartile ranges. IFN, interferon; IL, interleukin; CXCL10, C-X-C motif chemokine ligand 10; VEGF, vascular endothelial growth factor; NLR, neutrophil-to-lymphocyte ratio; LD, lactate dehydrogenase; CRP, C-reactive protein; **P*<0.05; ns, not significant.

**
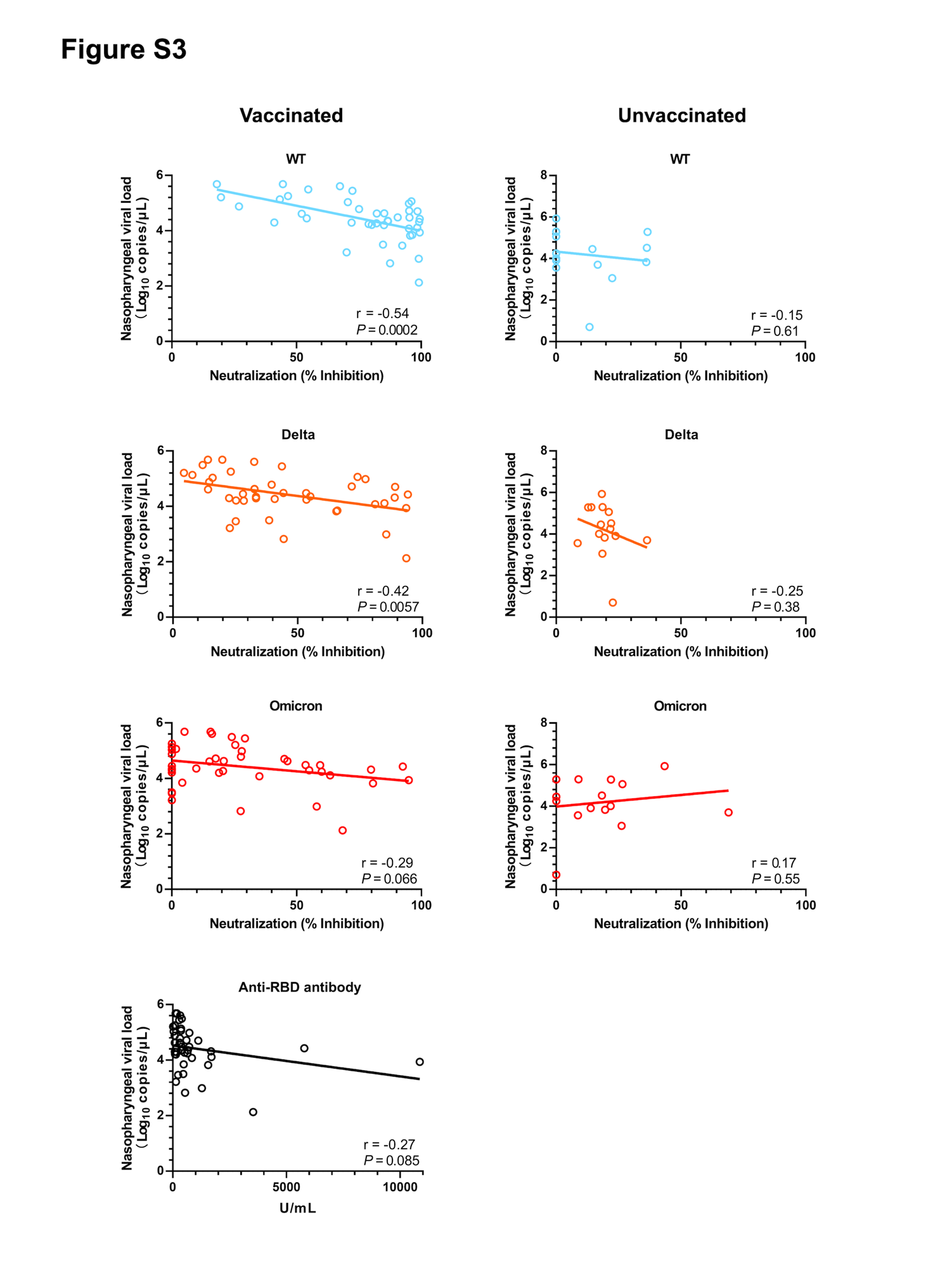
Figure S3.** Relationship between the initial nasopharyngeal viral load and the neutralization activity against the wild-type-, Delta-, Omicron-derived variants, and anti-RBD antibody levels in the vaccinated (n = 42) (left column) and unvaccinated patients (n = 15) (right column). The Pearson correlation was calculated, and the *P* value and r value are shown. The general linear regression lines are shown. Each level was measured at admission (within 5 days after symptom onset) and each dot represents an individual value. WT, wild-type; RBD, receptor-binding domain.

**
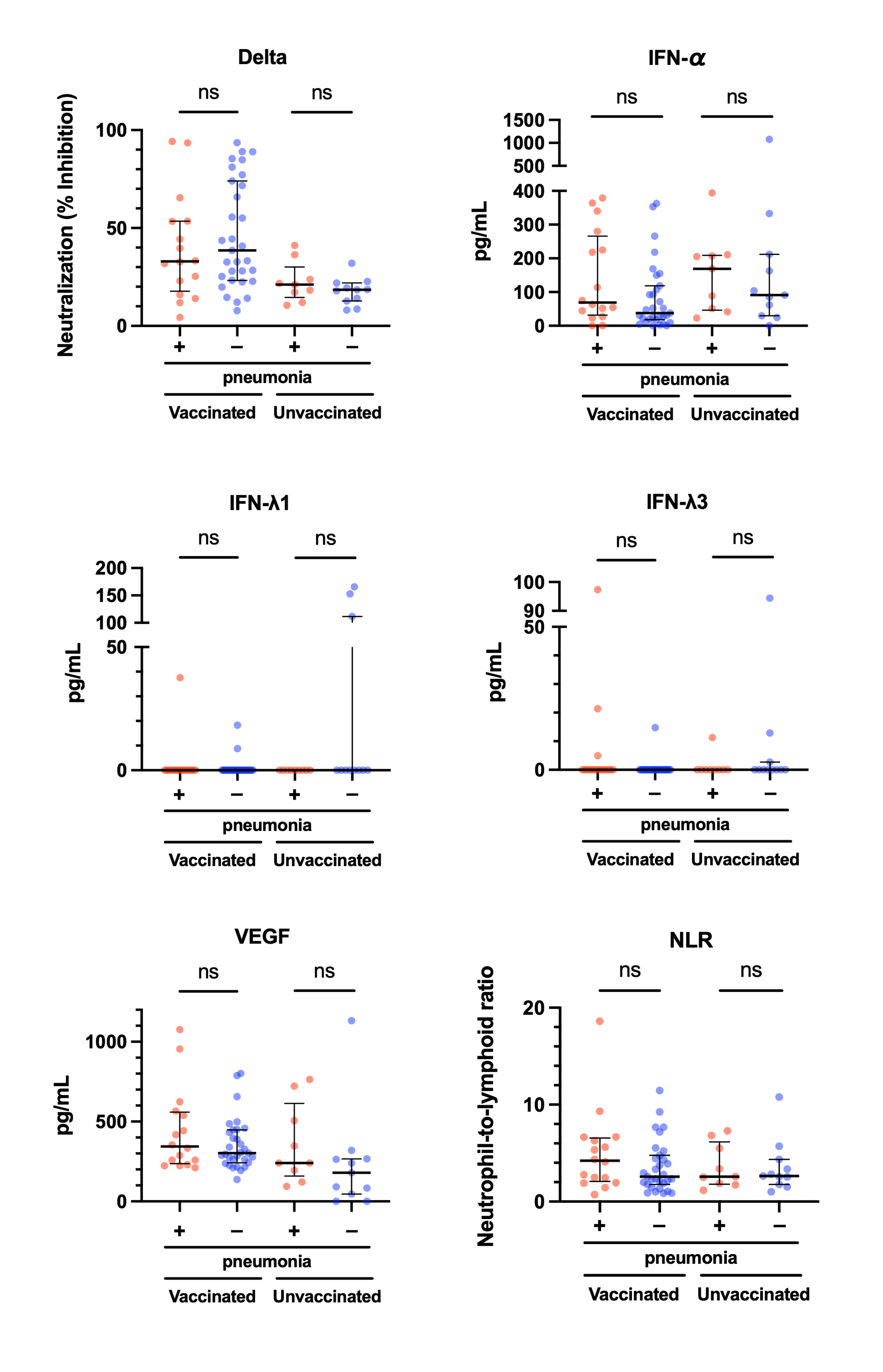
**

**Figure S4.** Relationship between the neutralizing activity against the Delta-derived variant or serum biomarker levels and the present of pneumonia at the acute phase of SARS-CoV-2 infection in vaccinated (n = 47) and unvaccinated patients (n = 20). The Mann-Whitney test was used to compare values between vaccinated patients with (n = 16) and without pneumonia (n = 31), and unvaccinated patients with (n = 9) and without pneumonia (n = 11). Each level was measured at admission (within 5 days after symptom onset) and each dot represents an individual value. IFN, interferon; VEGF, vascular endothelial growth factor; NLR, neutrophil-to-lymphocyte ratio; ns, not significant.
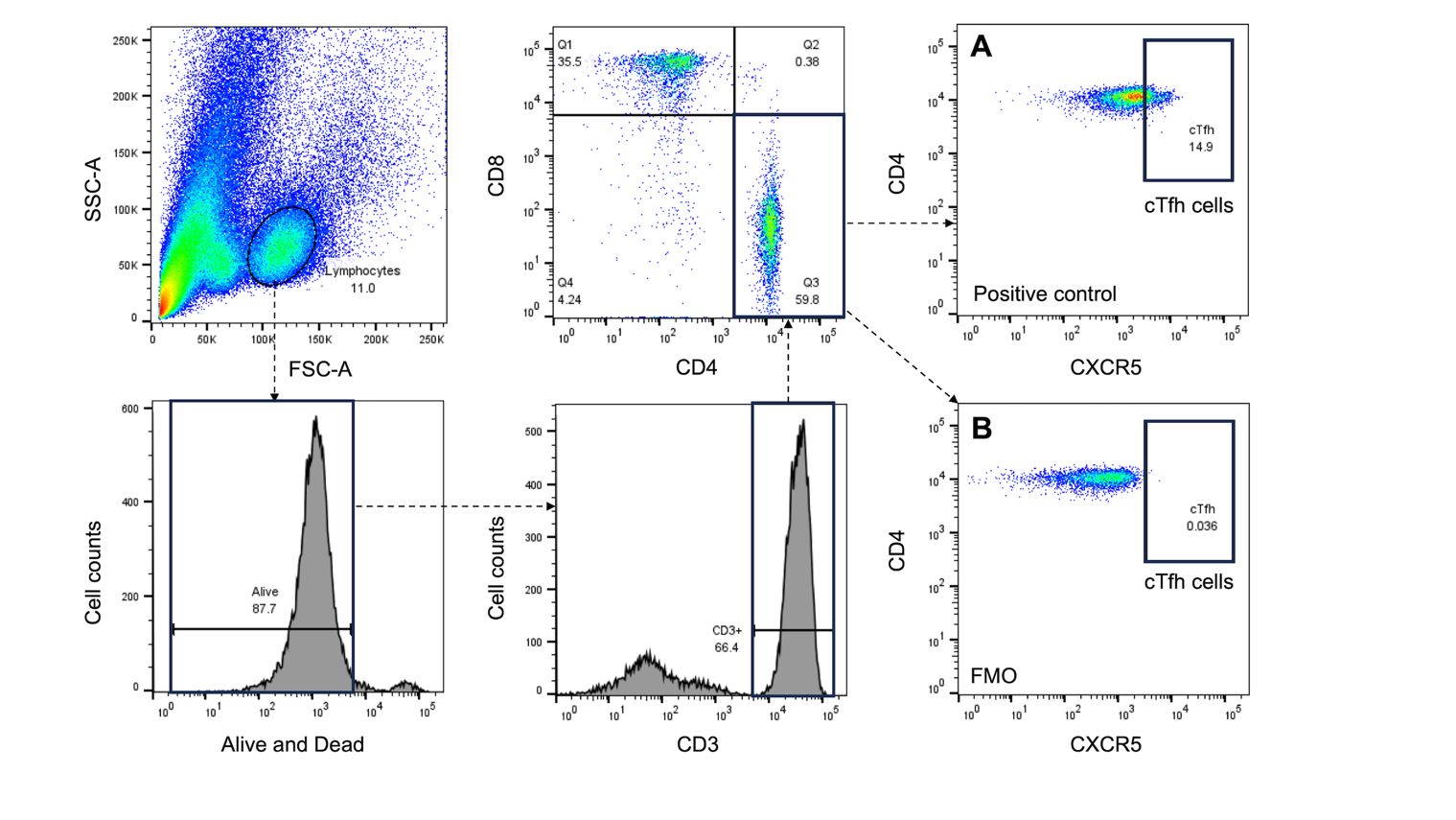
**Figure S5.** Flow cytometry plots of the gating strategy used to identify CXCR5^+^CD4^+^ cTfh cells. (A) Peripheral blood mononuclear cells (PBMCs) were isolated from a healthy individual who had received three doses of COVID-19 vaccination two weeks prior and then used as an internal positive control. (B) A fluorescence minus one (FMO) control was used for gating analyses to distinguish positively from negatively stained cell populations.

**Table S1.** List of flow cytometry antibodies used in this study

| **Parameter** | **Fluorochrome** | **Clone** | **Manufacturer** |
| --- | --- | --- | --- |
| CD3 | PE | UCHT1 | Biolegend (San Diego, CA, USA) |
| CD4 | FITC | RPA-T4 |  |
| CD8 | APC | SK1 |  |
| CXCR5 | PE-Cy7 | J252D4 |  |
| Live and Dead | APC-Cy7 | – |  |

**Table S2.** List of enzyme-linked immunosorbent assay kits used in this study

| **Immune indicators** | **Kit Name** | **Manufacturer** |
| --- | --- | --- |
| IFN-α | VeriKine-HS Human IFN Alpha All Subtype ELISA Kit | PBL Assay Science (Piscataway, NJ, USA) |
| IFN-λ1 (IL-29) | IL-29 Human ELISA Kit | Invitrogen (Waltham, MA, USA) |
| IFN-λ3 (IL-28B) | AuthentiKine Human IL-28B ELISA Kit | Proteintech (Rosemont, IL, USA) |
| IL-6 | AuthentiKine Human IL-6 ELISA Kit |  |
| CXCL10 | Human CXCL10/IP-10 ELISA Kit |  |
| VEGF | AuthentiKine Human VEGF ELISA Kit |  |
